# Wall blocks for breast cancer in pregnant patients: saving general anaesthesia also benefits foetal wellness

**DOI:** 10.1186/s44158-025-00281-8

**Published:** 2025-10-09

**Authors:** Cristiano D’Errico, Annamaria Fabozzi, Giuseppe Sepolvere, Martino Trunfio, Michele Liguori, Cristina Manetti, Dario Paolo Anceschi, Raffaella Amato

**Affiliations:** 1 Department of Anaethesia and Intensive Care Unit, Nuovo Ospedale Della Costiera, Sorrento, Naples, Italy; 2 Department of Obstretics and Ginecology, Santa Maria Delle Grazie Hospital, Pozzuoli, Naples, Italy; 3 Department of Anaesthesia and Cardiac Surgery Intensive Care Unit, San Michele Hospital, Maddaloni, Caserta, Italy; 4 Department of Breast Unit- A.O.R.N. “Cardarelli”, Naples, Italy; 5 Federico II University, Naples, Italy

**Keywords:** Fascial plane block, Pregnancy

## Abstract

Although it is uncommon in general, breast cancer is the most commonly diagnosed cancer during pregnancy. Pregnant patients should receive treatment based on nonpregnant guidelines, with special adjustments for diagnosis, staging, oncology, and obstetrics. This situation is particularly concerning for the health of a long-awaited foetus, especially after medical intervention to aid fertilization. To ensure the baby's safety, it is best to conclude the pregnancy as soon as possible in many cases. We know this is not always possible. This case report discusses the application of the pecto-serratus plane block (PSP) in a patient at seven months gestation undergoing breast quadrantectomy due to the abrupt onset of breast cancer. This study is limited as it involves only one patient. However, it highlights the relevance of locoregional anaesthesia in para-physiological states such as pregnancy.

## Introduction

Although it is uncommon in general, breast cancer is the most commonly diagnosed cancer during pregnancy [1]. Treatment for pregnant patients should follow nonpregnant patient guidelines, with specific considerations for diagnosis, staging, oncological treatment, and obstetrical care. About 7 to 10% of women diagnosed with breast cancer are under 40. This subgroup of patients has different risk factors, cancer biology, clinical outcomes, and specific psychosocial issues, such as fertility preservation, family planning, and job reintegration [2]. Early full-term pregnancy has been shown to reduce the lifetime risk of breast cancer, whereas a later first full-term pregnancy increases the risk of breast cancer. This should be considered to assess the timing of intervention.

The best time for surgery during pregnancy is the second trimester due to lower risks for both the foetus and preterm labour.

The anaesthesia technique depends on the procedure duration, gestational age, and mother and child condition [3]. Surgery for breast cancer during pregnancy follows treatment guidelines and is performed in any trimester regardless of anaesthesia preferences. Fascia blocks are considered a suitable choice today due to their benefits on the hypothalamic‒pituitary‒adrenal axis, which may reduce relapse risks for the mother and their impact on the health of the unborn child by eliminating the risk of transplacental passage of anaesthetics. Anaesthetic agents commonly used for surgical procedures during pregnancy, such as barbiturates, propofol, benzodiazepines, opioids, and ketamine, do not show teratogenic effects when administered at clinical doses. Considering that foetal blood concentrations of muscle relaxants are only 10% to 20% of maternal concentrations, these drugs appear to have a substantial margin of safety when given to pregnant women during organogenesis. There is no evidence of teratogenicity with the clinical administration of local anaesthetic agents. Only maternal cocaine abuse is associated with congenital cardiac defects and facial, genitourinary, and gastrointestinal tract anomalies. Given that anaesthetics are currently deemed safe for unborn children, do you prefer to ensure your child’s health by avoiding any drugs passing through the placenta?

Considering the recent concerns regarding fascia blockage in oncological surgery [4], it is advisable, in cases of pregnancy complicated by breast cancer, to avoid general anaesthesia for pregnant patients. Whenever feasible, locoregional anaesthesia should be practised.

To execute this block, “written” consent was obtained from the patient for the publication of this case report.

## Case overview

**Fig. 1:**
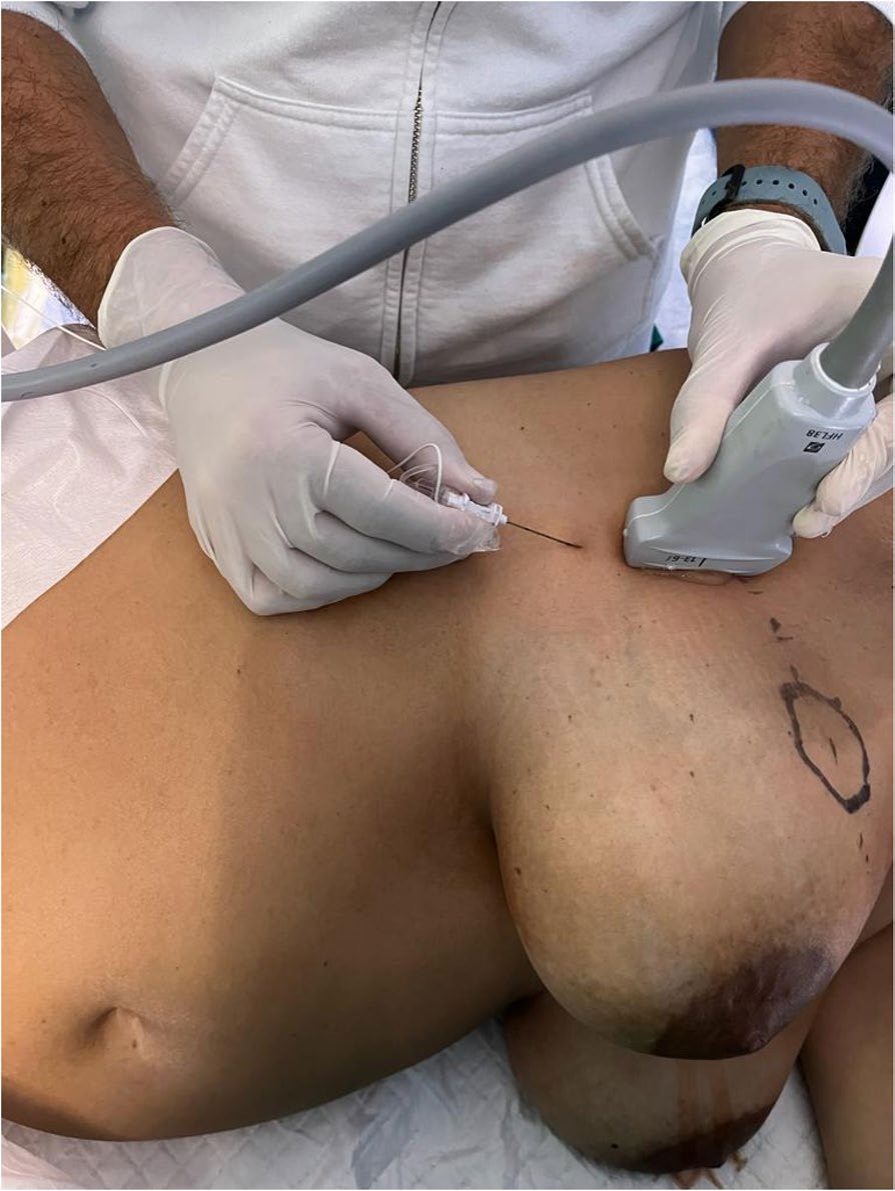
Serratus plane block is performed to a pregnant woman

**Fig. 2:**
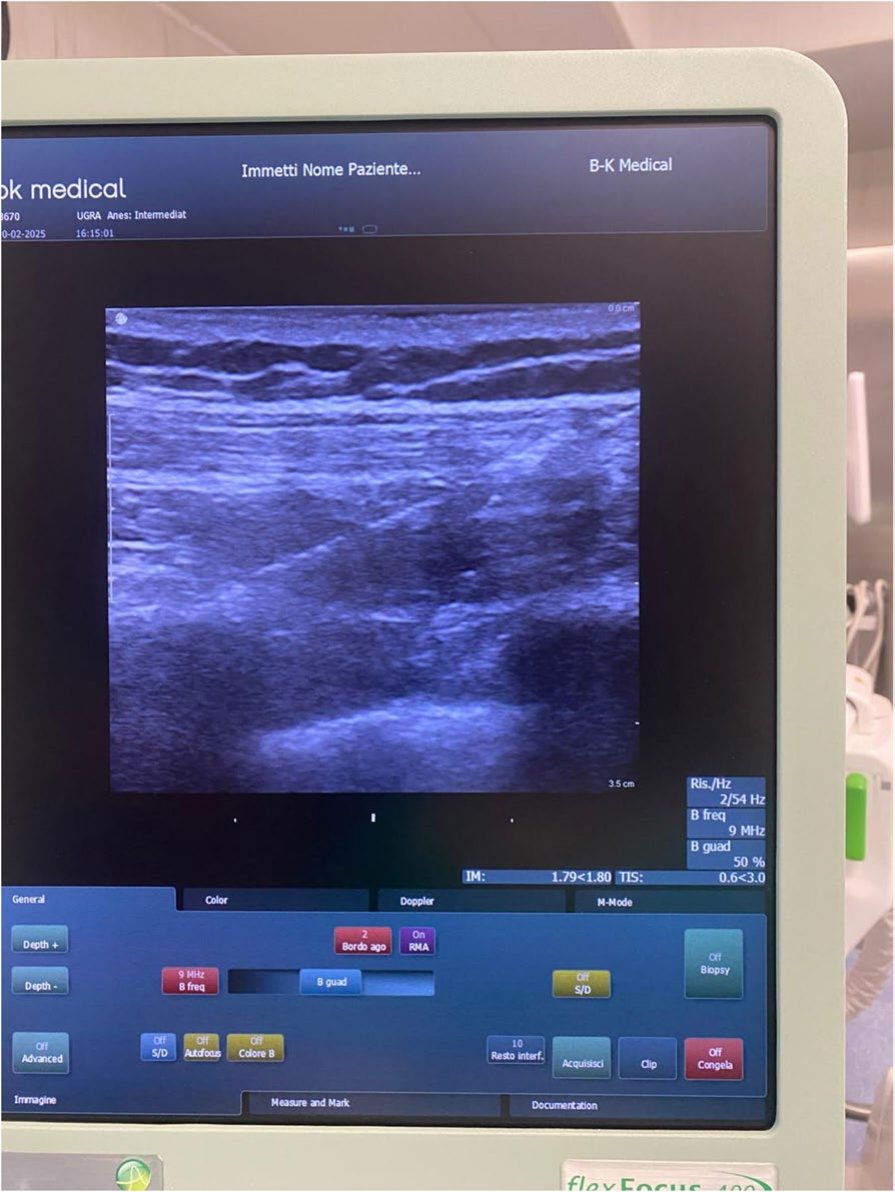
The needle in contact with the fourth rib in the pectoserratus plane

We present a case of a Pectoserratus plane (PSP) block in a 45-year-old pregnant woman following the AMC technique. The patient had a quadrantectomy for a 2.2 cm cancer in the right upper outer quadrant and removal of the sentinel lymph node (Figs. 1 and 2). With no other pathologies, her ASA classification was I.

**Fig. 3:**
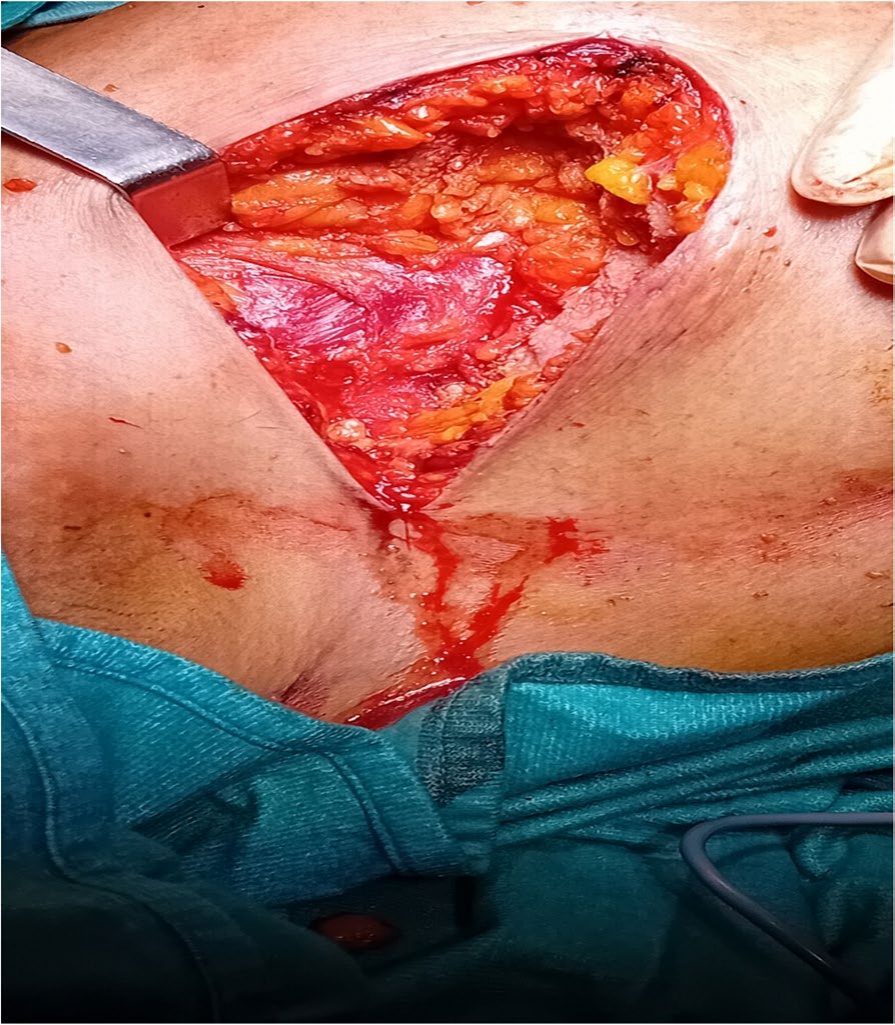
Upper left quadrantectomy

A 100-mm 22 G needle was used for the PSP block, and 3 ml of NaCl (sodium chloride) was initially injected to confirm placement (Fig. 3). Based on the presence of the Double-V sign, 150 mg of ropivacaine 0.75% combined with dexamethasone 2 mg was administered.

Local anaesthetics spread evenly and dynamically through the fascia.

The patient was lightly sedated with 0.03 mg/kg midazolam and di-isopropylphenol via a targeted controlled infusion of 1 mcg/ml and spontaneously breathed with nasal cannulas. No opioids were administered.

No haemodynamic changes were reported, and no signs or symptoms associated with brain lesions were detected. The patient was prematurely discharged from the hospital within the next eight hours; only intravenously, 1 g of paracetamol was prescribed as a numeric rating scale (NRS) < 2.

Foetal echocardiography was done before and after the intervention.

## Discussion

The use of a loco-regional anaesthesia technique is now widely used in breast surgery. Although it is equally well known how the transplacental passage of drugs for general anaesthesia can put at risk the newborn, there are no works in the literature that have highlighted the use of loco-regional anaesthesia for breast cancer. This case report highlights the importance of fascia blocks in pregnant women.

## Data Availability

No datasets were generated or analysed during the current study.

## Glossary

PSP: Pecto-serratus plane block
NaCl: Sodium chloride
AMC: Assisted medical creation
